# *Astragalus mongholicus Bunge* Water Extract Exhibits Anti-inflammatory Effects in Human Neutrophils and Alleviates Imiquimod-Induced Psoriasis-Like Skin Inflammation in Mice

**DOI:** 10.3389/fphar.2021.762829

**Published:** 2021-12-10

**Authors:** Wei-Jen Cheng, Chih-Chao Chiang, Cheng-Yu Lin, Yu-Li Chen, Yann-Lii Leu, Jia-Yu Sie, Wen-Ling Chen, Chung-Yuan Hsu, Jong-Jen Kuo, Tsong-Long Hwang

**Affiliations:** ^1^ Graduate Institute of Clinical Medical Sciences, College of Medicine, Chang Gung University, Taoyuan, Taiwan; ^2^ School of Traditional Chinese Medicine, College of Medicine, Chang Gung University, Taoyuan, Taiwan; ^3^ Center for Traditional Chinese Medicine, Chang Gung Memorial Hospital, Taoyuan, Taiwan; ^4^ Puxin Fengze Chinese Medicine Clinic, Taoyuan, Taiwan; ^5^ Graduate Institute of Natural Products, College of Medicine, Chang Gung University, Taoyuan, Taiwan; ^6^ Research Center for Chinese Herbal Medicine, Research Center for Food and Cosmetic Safety, Graduate Institute of Health Industry Technology, College of Human Ecology, Chang Gung University of Science and Technology, Taoyuan, Taiwan; ^7^ Graduate Institute of Traditional Chinese Medicine, School of Traditional Chinese Medicine, College of Medicine, Chang Gung University, Taoyuan, Taiwan; ^8^ Department of Anesthesiology, Chang Gung Memorial Hospital, Taoyuan, Taiwan; ^9^ Department of Chemical Engineering, Ming Chi University of Technology, New Taipei City, Taiwan

**Keywords:** neutrophil, inflammation, psoriasis, traditional Chinese medicine, *astragalus mongholicus bunge*

## Abstract

Neutrophils are the primary immune cells in innate immunity, which are related to various inflammatory diseases. *Astragalus mongholicus Bunge* is a Chinese medicinal herb used to treat various oxidative stress-related inflammatory diseases. However, there are limited studies that elucidate the effects of *Astragalus mongholicus Bunge* in human neutrophils. In this study, we used isolated human neutrophils activated by various stimulants to investigate the anti-inflammatory effects of *Astragalus mongholicus Bunge* water extract (AWE). Cell-free assays were used to examine free radicals scavenging capabilities on superoxide anion, reactive oxygen species (ROS), and nitrogen-centered radicals. Imiquimod (IMQ) induced psoriasis-like skin inflammation mouse model was used for investigating anti-psoriatic effects. We found that AWE inhibited superoxide anion production, ROS generation, and elastase release in human neutrophils, which exhibiting a direct anti-neutrophil effect. Moreover, AWE exerted a ROS scavenging ability in the 2,2’-Azobis (2-amidinopropane) dihydrochloride assay, but not superoxide anion in the xanthine/xanthine oxidase assay, suggesting that AWE exhibited anti-oxidation and anti-inflammatory capabilities by both scavenging ROS and by directly inhibiting neutrophil activation. AWE also reduced CD11b expression and adhesion to endothelial cells in activated human neutrophils. Meanwhile, in mice with psoriasis-like skin inflammation, administration of topical AWE reduced both the affected area and the severity index score. It inhibited neutrophil infiltration, myeloperoxidase release, ROS-induced damage, and skin proliferation. In summary, AWE exhibited direct anti-inflammatory effects by inhibiting neutrophil activation and anti-psoriatic effects in mice with IMQ-induced psoriasis-like skin inflammation. Therefore, AWE could potentially be a pharmaceutical Chinese herbal medicine to inhibit neutrophilic inflammation for anti-psoriasis.

## 1 Introduction

*Astragalus mongholicus Bunge* (AM; “Huang Qi” in Chinese) is one of the most important and widely used traditional Chinese medicines, having anti-inflammatory effects and multiple bioactivities such as protecting viral myocarditis injury, regulating blood sugar, and promoting wound healing (Chen et al., 2012; Agyemang et al., 2013; Piao and Liang, 2014; Qi et al., 2017). Furthermore, AM is also an antioxidant with a mitochondrial protective effect that contributes to anti-aging and anti-cancer effects (Li XT. et al., 2012). AM contains several bioactive ingredients, such as polysaccharides, flavonoids, and saponins (Fu J. et al., 2014), that are reported to provide anti-inflammatory effects and other benefits against many diseases or pathological conditions in various models (Shahzad et al., 2016).

Neutrophils are the most abundant leukocytes for innate immunity in the human body. In the process of respiratory burst, activated neutrophils generate abundant superoxide anion via reduced nicotinamide adenine dinucleotide phosphate (NADPH) oxidase (Dahlgren and Karlsson, 1999; Jaquet et al., 2011; Wang et al., 2012; Ruhnau et al., 2014), which are converted to reactive oxygen species (ROS), such as OH^–^, HOCl, and H_2_O_2_, by catalase, myeloperoxidase (MPO), and superoxide dismutase (SOD) (Thannickal and Fanburg, 2000; Dupre-Crochet et al., 2013; Reczek and Chandel, 2015). As such, neutrophils tend to produce an enormous amount of ROS against microorganisms, including bacteria, fungi, and viruses. However, excessive production of ROS from neutrophils may cause tissue damage (Glennon-Alty et al., 2018). Aside from that, activated neutrophils also release granules containing proteolytic enzymes, such as neutrophil elastases, MPO, and cathelicidin (Faurschou and Borregaard, 2003), that contribute to tissue damage. Overproduction of ROS, proteolytic enzymes, and cytokines by neutrophils can also cause several pathological conditions, which could aggravate many clinical diseases (Papayannopoulos, 2018).

One such condition is psoriasis, an inflammatory skin disease with complicated etiology. Psoriasis reportedly affects approximately 3% of the population worldwide (Gupta et al., 2014). However, the prevalence of this disease varies with race and geographic location. For example, in a review of epidemiological data of psoriasis, the overall prevalence of psoriasis was estimated to be 3.7% in the US National Health Survey. However, studies in Asians revealed a prevalence ranging from 0.05 to 0.47% (Kaufman and Alexis, 2018). In Europe, the prevalence of psoriasis is approximately 2% according to a previous study (Boehncke and Schön, 2015). Moreover, psoriasis has been reported to affect 1.9–3.5% of people in eastern Africa, but only 0.025–0.9% in western Africa. This difference has been attribute to the genetic difference among different races and ethnicities (Kaufman and Alexis, 2018). Its classical manifestations include raised plaques and scales on the skin, and these psoriasis skin lesions are attributed to the over-proliferation and abnormal differentiation of keratinocytes, which is related to tissue damage by ROS and the release of cytokines by the infiltration of adaptive and innate immune cells (Rendon and Schäkel, 2019). Moreover, psoriatic lesions also usually exhibit neutrophil accumulation (Schön et al., 2017). Previous studies showed that neutrophils play a role in amplifying immune reaction, and neutrophil extracellular traps correlate with disease severity in patients with psoriasis (Lowes et al., 2014; Chiang et al., 2019). Oxidative stress is also involved in disease severity in clinical psoriasis patients (Cannavò et al., 2019). It influences the pathogenesis of psoriasis via several signaling pathways, such as nuclear factor-κB (NF-κB) and mitogen-activated protein kinase (MAPK) (Zhou et al., 2009). In the mouse model of imiquimod (IMQ)-induced psoriasis-like skin inflammation, excess ROS production contributed to histopathological changes in skin lesions (Baek et al., 2012).

Currently, T cells and dendritic cells are considered as the primary immune cells that release cytokines associated with psoriasis, such as interleukin (IL)-17A, IL-22, tumor necrosis factor (TNF)-α, and interferon (IFN)-γ, which affect keratinocyte proliferation (Nadeem et al., 2020b). Developing drugs that target these inflammatory mediators is one of the therapeutic strategies for psoriasis. For example, ibrutinib, a Bruton’s tyrosine kinase inhibitor, suppresses IMQ-induced psoriasis-like skin inflammation in mice by downregulating IL-17A and IL-23 expression (Nadeem et al., 2020a). It can also decrease oxidative stress and inflammatory mediator levels in neutrophils and dendritic cells (Al-Harbi et al., 2020). However, emerging evidence has demonstrated that neutrophils may play a crucial role in psoriasis (Wang and Jin, 2020). Neutrophils produce many inflammatory mediators, forming neutrophil extracellular traps (NETs) and secreting IL-17, contributing to psoriasis skin inflammation (Schön et al., 2017). It is well-known that the interaction between IL-17A and keratinocyte causes the pathogenesis of psoriasis (Furue et al., 2020). Nevertheless, neutrophils are the primary source of IL-17A expression observed in psoriatic skin lesions, and interactions between infiltrated neutrophils and keratinocytes strengthen the IL-23/Th17 axis (Katayama, 2018). Moreover, neutrophils and mast cells, not T cells, are the dominant immune cells that release IL-17 in psoriatic skin, and neutrophils release abundant IL-17 during NET formation (Lin et al., 2011). A study demonstrated that NETs generated by neutrophils could establish self-amplifying inflammation reactions in psoriasis (Herster et al., 2020). In summary, neutrophils could secrete IL-17A, which affects keratinocytes, after infiltrating into the epidermis. NET formation by neutrophils also amplifies the inflammatory reactions in innate and adaptive immune cells, leading to the pathogenesis of psoriasis (Chiang et al., 2019).

Despite reports that AM can exhibit anti-inflammatory and anti-oxidation effects against various conditions, whether it could offer the same benefits against psoriasis and ROS overproduction by neutrophils remains unknown. Herein, we investigate *Astragalus mongholicus Bunge* water extract (AWE) and its anti-inflammatory effect on human neutrophils. We also examined the potential therapeutic effect of topical AWE against psoriasis in mice.

## 2 Materials and Methods

### 2.1 Reagents

The herbal materials used in this study were the dried roots of *Astragalus mongholicus Bunge* which were purchased from Chang Gung Memorial Hospital, Taoyuan, Taiwan. Astragaloside IV was purchased from Sunhank technology Co. Ltd., Tainan, Taiwan. Calycosin-7-O-β-D-glucoside was purchased from Wuhan ChemFaces Biochemical Co., Ltd. (Wuhan, China). Ononin and calycosin were purchased from Sigma-Aldrich (St. Louis, MO, United States). Ficoll-paque PLUS was purchased from GE Healthcare (Little Chalfont, Buckinghamshire, United Kingdom). Hank’s balanced salts solution (HBSS) was purchased from Gibco (Grand Island, NY, United States). Meo-Sac-Ala-Ala-Pro-Val-*p*-nitroanilide was purchased from Calbiochem (La Jolla, CA, United States). Lactate dehydrogenase (LDH) assay kits were purchased from Promega (Madison, WI, United States). Leukotriene B4 (LTB_4_) and Leu-Glu-Ser-Ile-Phe-Arg-Ser-Leu-Leu-Phe-Arg-Val-Met (trifluoroacetate salt, MMK-1) were purchased from Tocris Bioscience (Ellisville, MO, United States). Xanthine was purchased from Santa Cruz Biotechnology (Santa Cruz, CA, United States). Water-soluble tetrazolium-1 (WST-1) was obtained from Dojindo (Kumamoto, Japan). Dihydroethidine (HE) was purchased from Invitrogen (Carlsbad, CA, United States). Fluorescein isothiocyanate (FITC)-labeled anti-cluster of differentiation molecule 11b (CD11b) was purchased from eBioscience (San Diego, CA, United States). Anti-lymphocyte antigen 6 complex locus G6D (Ly6G) antibody was purchased from BioLegend (San Diego, CA, United States). Anti-myeloperoxidase (MPO) antibody was obtained from Abcam (Cambridge, United Kingdom). Anti-Ki67 antibody was procured from GeneTex International Corporation (Hsinchu, Taiwan). Anti-4-Hydroxynonenal (HNE) antibody was purchased from Bioss Antibodies Inc. (Woburn, MA, United States). The rabbit anti-rat immunoglobulin G (IgG) was from ImmunoReagent Inc. (Beijing, China). Other bioassay and reagents were purchased from Sigma-Aldrich (St. Louis, MO, United States).

### 2.2 Astragalus mongholicus Bunge Water Extract Preparation

AWE was prepared in boiling water. Briefly, 131.25 g (35 Taiwan mace) of herbal materials was soaked in 600 ml of sterile double distilled water (ddH_2_O) for 30 min and then boiled at 100°C for another 30 min. The extracts were filtered through a sterile 0.45 µm filter and then concentrated and powdered with an oil-pumping freeze dryer (LABCONCO, Kansas City, MO, United States). The AWE powder was stored at –20°C until further use and was dissolved in ddH_2_O before use in the subsequent experiments. A voucher specimen (CGU_AM-01) was deposited in the Graduate Institute of Natural Products, Chang Gung University, Taoyuan, Taiwan.

### 2.3 High-Performance Liquid Chromatography Analysis

A Hitachi chromatography system (Hitachi, Tokyo, Japan) was used to analyze the composition of AWE. A previous study described the setting of the HPLC machine, standard solution preparation, and AWE solution preparation in detail (Matkowski et al., 2003). Ononin, calycosin, and calycosin-7-O-β-D-glucoside were used as standard references. AWE fingerprinting was performed under the detection wavelength at 260 nm.

### 2.4 Ultra-Performance Liquid Chromatography-Tandem Mass Spectrometry Condition

A Shimazu UPLC-MS/MS system coupled with Nexera X2 UPLC and LCMS-8045 (Shimazu, Kyoto, Japan) was used to analyze Astragaloside IV, the marker constituent of AWE according to government guidelines of Taiwan Herbal Pharmacopeia. Liquid chromatography was carried out using a YMC-Triart C18 column (2.1 × 50 mm, 1.9 μm particle size) (YMC, Kyoto, Japan). The mobile phase was 34% MeCN aqueous solution with 0.1% formic acid. The injection volume was 5 μL. The flow rate was 0.5 ml/min. The column temperature was maintained at 40°C. For the sample preparation, 110 mg AWE was dissolved in 10 ml of ddH_2_O and partitioned with 10 ml of *n*-Butanol three times. Before analysis, the organic layer (18.4 mg) was collected and dissolved in MeOH at a 1 mg/ml concentration (Kafle et al., 2020).

### 2.5 Human Neutrophil Isolation

This study was approved by the institutional review board of Chang Gung Memorial Hospital (Registration number: IRB 100 1278C). All healthy volunteers aged 20–32 years old provided written consent prior to blood donation. Human neutrophils were prepared and isolated from healthy individuals using the Ficoll-Hypaque density gradient centrifugation method (Paoliello-Paschoalato et al., 2014). The isolated human neutrophils were suspended at 4°C in Ca^2+^-free Hanks’ balanced salt solution (HBSS) before further experiments (Tsai et al., 2015).

### 2.6 Reactive Oxygen Species Measurement

#### 2.6.1 Detecting Extracellular Superoxide Anion Production

Human neutrophils (3 × 10^5^ or 6 × 10^5^ cells/mL) were suspended in 1 mM CaCl_2_ and 0.5 mg/ml ferricytochrome *c* at 37°C for 5 min, and then incubated with ddH_2_O (as the control) or AWE (10, 30, or 100 µg/ml) for another 5 min. After which, neutrophils were activated by *N*-formyl-L-methionyl-L-leucyl-L-phenylalanine (fMLF, a formyl peptide receptor (FPR) one agonist, 0.1 µM for 10 min), MMK-1 (a FPR2 agonist, 0.1 µM for 10 min) pre-treated with cytochalasin B (CB, 1 µg/ml) for 3 min, or phorbol 12-myristate 13-acetate (PMA, a protein kinase C (PKC) activator, 10 nM for 5 min). The reduced ferricytochrome *c* was detected at 550 nm using a spectrophotometer (Hitachi, U-3010, Tokyo) (Chiang et al., 2020).

#### 2.6.2 Detecting Total ROS Production

Intracellular and extracellular ROS generated by activated human neutrophils were detected by the luminol method as luminol releases a chemiluminescent signal when reacted with non-specified ROS that is produced by activated human neutrophils. For this analysis, human neutrophils (7 × 10^5^ cells/mL) were mixed with 37.5 µM luminol and horseradish peroxidase (HRP) for 5 min to enhance the reaction, and then ddH_2_O (as the control) or AWE (10, 30, or 100 µg/ml) was added. After 5 min, neutrophils were activated either by fMLF (0.1 µM) for 5 min or by PMA (10 nM) for 30 min. A 96-well luminescence microplate reader (Tecan, Infinite F200 Pro, Tecan Group, Männedorf, Switzerland) was used to detect the chemiluminescence (Tsai et al., 2015).

#### 2.6.3 Detecting Intracellular Superoxide Anion Production

Human neutrophils (1 × 10^6^ cells/mL) were pre-incubated with Hydroethidine bromide (HE; 1 μM), a superoxide anion indicator that can pass through the cell membrane, for 10 min at 37°C, and then washed out, so that the neutrophils were labeled with HE intracellularly. Thereafter, ddH_2_O (as the control) or AWE (10, 30, or 100 µg/ml) was added 5 min before activating the neutrophils with 0.1 µM fMLF with 3 min CB (0.5 µg/ml) pre-treatment or 10 nM PMA for 5 min. The intracellular fluorescence intensities of HE were detected using flow cytometry (Yang et al., 2018).

### 2.7 Measurement of Elastase Release for Neutrophil Degranulation

Meo-Sac-Ala-Ala-Pro-Val-*p*-nitroanilide is a neutrophil elastase-specified substrate. The method used to detect elastase release, which represented the degranulation of azurophilic granules by activated neutrophils, was previously described (Lee CL. et al., 2013). Human neutrophils (6 × 10^5^ or 1 × 10^6^ cells/mL) were pre-incubated with 1 mM CaCl_2_ and 0.1 mM substrate at 37°C for 5 min, and then we added ddH_2_O (as the control) or AWE (10, 30, or 100 µg/ml), and then incubated for another 5 min. Human neutrophils were stimulated by different activators, including fMLF (0.1 µM), MMK-1 (0.1 µM), leukotriene B4 (LTB_4_, 0.1 µM), or interleukin 8 (IL-8, 100 ng/ml) for 10 min with pre-treatment with CB (0.5 or 2 µg/ml) for 3 min. The substrate absorbance changes were detected spectrophotometrically at 405 nm to evaluate elastase release.

### 2.8 Lactate Dehydrogenase Assay for Cell Viability

Since lactate dehydrogenase (LDH) gets released when chemical agents cause cell death, it was used to assess the cytotoxicity of drugs. Human neutrophils (6 × 10^5^ cells/mL) in 1 mM CaCl_2_ were incubated with ddH_2_O (as the control) or AWE (10, 30, or 100 µg/ml) for 15 min, and then the cytotoxicity of AWE was determined by measuring LDH release at 492 nm. The total cell LDH was determined by lysing neutrophils with Triton X-100 (0.1%) at 37°C for 30 min. The protocol to detect LDH release has previously been described (Chang et al., 2008).

### 2.9 Antioxidant Capability Evaluation

#### 2.9.1 Superoxide Anion Scavenging Ability Analysis

Several cell-free systems were used to evaluate the antioxidant capability of AWE. The superoxide scavenging ability of AWE was analyzed by superoxide anions which generated by the xanthine/xanthine oxidase catalysis reaction. This reaction was facilitated by mixing Tris buffer (pH 7.4) in 1 mM CaCl_2_ with WST-1 (0.3 mM) and xanthine oxidase (0.02 U/mL) at 30°C, and then ddH_2_O (as the control), 20 U/ml superoxide dismutase (SOD, as the positive control) or AWE (10, 30, or 100 µg/ml) was added for 3 min. Then xanthine was added for 10 min. The amount of reduced WST-1 by superoxide anion was measured by the change in absorbance at 450 nm (Lin et al., 2009).

#### 2.9.2 ROS Scavenging Ability Analysis

We used fluorescence to detect ROS production by 2,2’-azobis (2-amidinopropane) dihydrochloride (AAPH), a water-soluble free radical-generating azo compound, for evaluating the ROS scavenging ability of AWE since ROS is generated when AAPH is dissolved in water. Fluorescein (80 nM) in 75 mM sodium phosphate buffer (pH 7.4) was incubated with ddH_2_O (as the control), Trolox (as the positive control), or AWE (10, 30, or 100 µg/ml) at 37°C for 5 min. After adding AAPH (25 mM), the changes in fluorescence intensity were monitored every 3 min for 120 min. Following the methods by a previous study (Betigeri et al., 2005), the excitation and emission wavelengths used were 485 and 535 nm, respectively.

#### 2.9.3 Nitrogen-Centered Radicals Scavenging Ability Analysis

We dissolved 1,1-diphenyl-2-picrylhydrazyl (DPPH; 100 μM), a stable nitrogen-centered free radical organic compound, in 99% ethanol, and then added ddH_2_O (as the control), α-tocopherol [vitamin E (3, 15, and 30 μM), as the positive control], or AWE (10, 30, or 100 µg/ml), and incubated the assay at 25°C for 15 min. The absorbance change was measured spectrophotometrically at 517 nm (Chen et al., 2018).

Another nitrogen-centered free radical compound, 2,2’-azino-bis(3-ethylbenzothiazolin-6-sulfonic acid) diammonium salt (ABTS), which has a blue color when dissolved in a potassium persulfate solution, was used in this study. ABTS (7 mM) was mixed with potassium persulfate (2.45 mM) in ddH_2_O, and then ddH_2_O (as the control), L-ascorbic acid (vitamin C (10, 15, and 30 μM), as the positive control), or AWE (10, 30, or 100 µg/ml) was added, then the assay was incubated at 25°C for 15 min. The change in light absorbance was determined spectrophotometrically at 734 nm (Rajendran et al., 2004).

### 2.10 Assessment CD11b Expression in Activated Neutrophils

Integrins, a kind of membrane protein, which promotes neutrophils to adhere to endothelial cells and then migrate to the inflammatory site, were expressed in activated neutrophils. In this study, we evaluated the effect of AWE on CD11b expression in fMLF-activated neutrophils. Human neutrophils (2.5 × 10^6^ cells/ml) were incubated with ddH_2_O (as the control) or AWE (10, 30, or 100 µg/ml) at 37°C for 5 min, and then neutrophils were stimulated by fMLF (0.1 μM) for another 5 min (pre-treated with 1 μg/ml CB for 3 min). The supernatant was removed after centrifugation (200 g, for 8 min) at 4°C, then neutrophils in the pallet were resuspended in HBSS, which contained 0.5% bovine serum albumin (BSA) and FITC-labeled anti-CD11b (1 μg/ml) and incubated at 4°C for 90 min in the dark. Immunofluorescence was measured using flow cytometry (Chiang et al., 2020).

### 2.11 Functional Study of Neutrophil Adhesion

Since neutrophil adhesion to endothelial cells plays a key role in the inflammatory response, the inhibition of adhesion will influence the recruitment of neutrophils to the inflammatory site. To assess the effect of AWE on neutrophil adhesion function *in vitro*, we used the mouse brain-derived endothelial cell 3 (bEnd.3). Human neutrophils (4 × 10^6^ cells/mL) were pre-treated with Hoechst 3,342 (1 ng/ml) at 37°C for 10 min. After washing out the dye by centrifugation (200 × g, for 8 min) and removing the supernatant, neutrophils were resuspended and incubated with ddH_2_O or AWE (100 µg/ml) for 5 min, then were activated with fMLF (0.1 μM) for 10 min, with pre-treatment with CB (1 μg/ml) for 2 min. Activated neutrophils were transferred to wells containing bEnd.3 cells and incubated for 15 min at 37°C. After incubation, the medium and non-adherent neutrophils were washed with HBSS, and the adherent neutrophils were fixed with 4% paraformaldehyde in HBSS. Cells were observed and counted using a fluorescence microscope (IX81; Olympus, Center Valley, PA, United States) (Chen et al., 2016).

### 2.12 Imiquimod-Induced Psoriasis-like Skin Inflammation in Mice

All animal experiments in this study were approved and regulated by the Institutional Animal Care and Use Committee of Chang Gung University, Taoyuan, Taiwan (IACUC approval No. CGU14-150).

Eight-to ten-week-old BALB/C male mice that weighed approximately 20 g each were obtained from BioLasco Co., Ltd., Yilan, Taiwan. Aldara (3M Health Care Limited, United Kingdom), which contains 5% imiquimod (IMQ), was used to induce psoriasis-like skin inflammation. AWE was dissolved in the vehicle with 40% ethanol and 60% ddH_2_O before application on the skin. The mice were divided into three groups: mice in the control group received no treatment; mice in the IMQ plus vehicle group were treated with IMQ (62.5 mg/day) and vehicle (100 µL/day); mice in the IMQ plus AWE group received equal doses of IMQ and AWE (10 mg/day), which were dissolved in vehicle (Chiang et al., 2020).

Before experiments, mice were given 1 week to adapt to the environment in 12-h light/12-h dark cycles with adequate food and water supply. The backs of mice were shaved with a depilatory machine and hair removal cream before the study. From days 1–5, 100 µL AWE or vehicle was topically applied to each mouse, followed by treatment with IMQ cream. Each day, we used pipette tips to drop 50 µL of AWE (100 mg/ml) or vehicle slowly and homogeneously on the back of the mice under anesthesia. After the solution had dried (approximately 10 min), we applied another 50 µL of solution on the back of the mice, that is, a total of 100 µL per day. We applied the IMQ cream approximately 30 min later while the solution had dried. Subsequently, we returned the mice to the cages and waited for them to awaken from anesthesia. A hand-held microscope and camera were used to obtain photos before the application of drugs every day. The severity of psoriatic lesions was assessed according to the human psoriasis area and severity index (PASI) score. The PASI score evaluates lesions including erythema, scaling, and thickness independently according to severity as: 0 for normal; one for mild; two for moderate; three for severe; four for very severe condition in psoriasis lesions. The images of psoriatic lesions were scored by two investigators, Dr. Cheng and Dr. Chiang. The image files were renamed for blinding purpose. The final scores of each image were the average score of the two investigators.

On day 6, mice were sacrificed by deep anesthesia, and then the skin samples were collected from their backs then fixed in 10% paraformaldehyde. The skin sections were subjected to hematoxylin–eosin (H&E) and immunohistochemical (IHC) staining including Ly6G (a neutrophil marker), MPO (an ROS catalyzer released by neutrophils), ki67 (a skin proliferation marker), and 4-HNE (an ROS-induced damage marker from lipid peroxidation chain reaction) following a standard protocol (Baek et al., 2012; El Malki et al., 2013). Images were quantified using ImageJ 1.53e (Wayne Rasband and contributors, Research Services Branch, National Institute of Health, Bethesda, Maryland, United States) (Schneider et al., 2012). The intensity and area of staining in IHC images were calculated. In HE images, epidermal area and average thickness were calculated.

### 2.13 Immunohistochemistry

The formalin-fixed paraffin-embedded skin samples were dewaxed and rehydrated before IHC. IHC was performed using an automatic immunohistochemistry staining device according to the manufacturer’s instructions (Bond, Leica BioSystems). The tissue sections were retrieved using Bond Epitope Retrieval Solution 1 (Ly6G, MPO, and 4-HNE) or 2 (Ki67) at 100°C for 20 min (Ly6G, MPO, and 4-HNE) or 30 min (Ki67) on the Bond-max automated immunostainer (Leica BioSystems) and stained with antibodies for Ly6G (mouse monoclonal antibody, 1:500 dilution; incubated for 60 min; BioLegend, category no. 127602), MPO (rabbit polyclonal antibody, 1:50 dilution; incubated for 60 min; Abcam, category no. ab9535), Ki67 (rabbit polyclonal antibody, 1:200 dilution; incubated for 30 min; GeneTex, category no.: GTX16667), and 4-HNE (rabbit polyclonal antibody, 1:600 dilution; incubated for 60 min; Bioss, category no.: bs-6313R). For Ly6G staining, secondary antibody staining with 5 µg/ml rabbit anti-rat IgG (ImmunoReagent, category no.: RbxRt-003-DHRPX) for 30 min was performed after primary staining. A polymer detection system (Bond Polymer Refine Detection, Leia BioSystems) was used to reduce nonspecific staining. The tissue sections were treated with liquid 3,3′-diaminobenzidine reagent using 3′-diaminobenzidine tetrahydrochloride as the chromogen and hematoxylin as the counterstain.

### 2.14 Statistical Analysis

All data were expressed as mean ± standard error of the mean (S.E.M.). One-way analysis of variance (ANOVA) was used for comparison between groups in this study. A two-tailed *p*-value < 0.05 represented a statistically significant difference. Post-hoc analyses were performed using Tukey’s multiple comparisons tests for each two-group comparison if the results of the one-way ANOVA indicated significant differences. Results with an adjusted *p*-value < 0.05 in Tukey’s multiple comparisons tests represented a statistically significant difference. For ordinal scale data analysis (PASI scores), a nonparametric analysis method of Kruskal–Wallis test was used in the ANOVA. Post-hoc multiple comparison analyses were performed using the two-stage linear step-up procedure of Benjamini, Krieger, and Yekutieli for every two-group comparison if the results of the Kruskal–Wallis test indicated significant differences. Results with an adjusted *q*-value < 0.05 using the two-stage linear step-up procedure of Benjamini, Krieger, and Yekutieli for multiple comparison tests represented a statistically significant difference. Data processing and analysis were conducted by using Sigmaplot 14.0 (Systat Software, San Jose, CA, United States) and Prism 9 (GraphPad Software, San Diego, CA, United States).

## 3 Results

### 3.1 Identification and Quantification of AWE Using HPLC and UPLC-MS/MS

**FIGURE 1:**
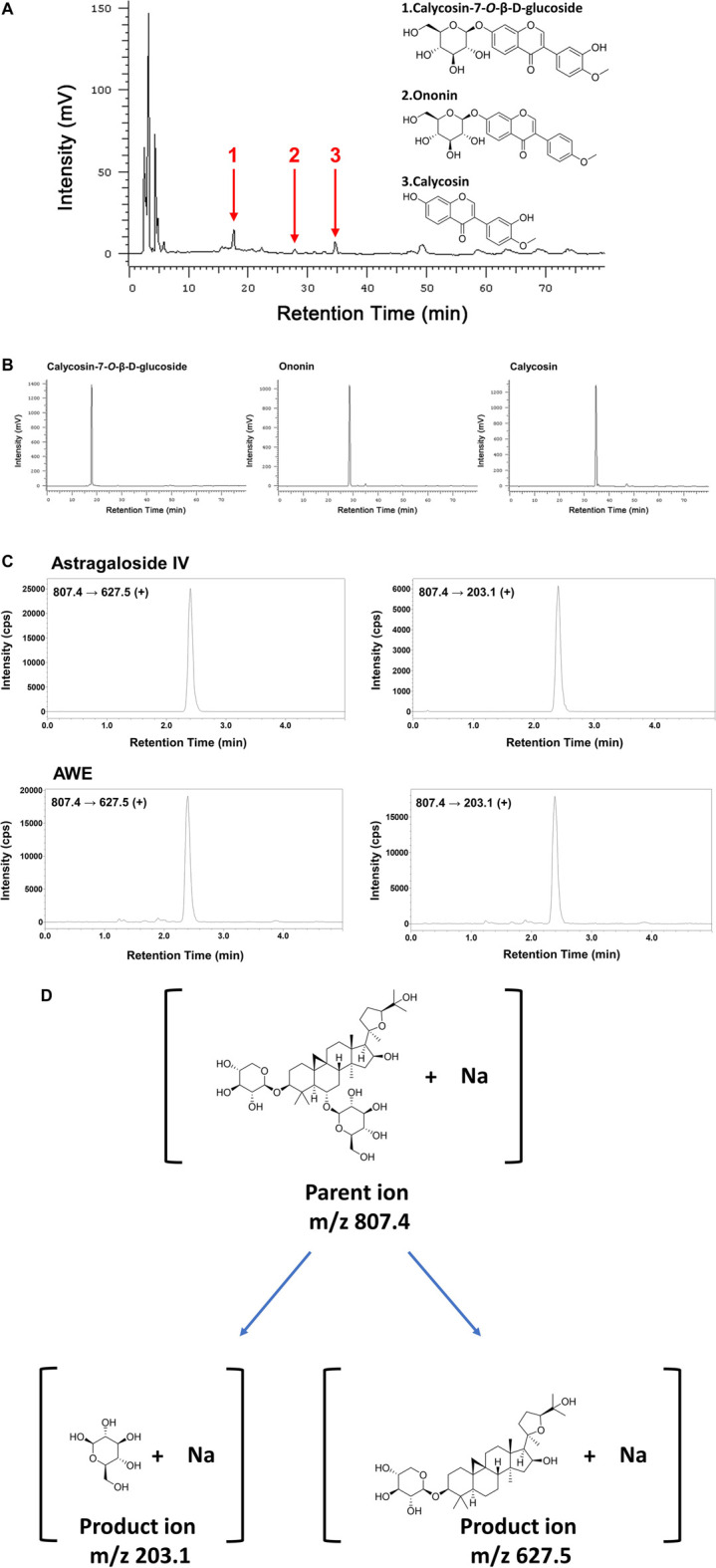
The chemical fingerprint of Astragalus mongholicus Bunge water extract (AWE). Chromatograms were obtained from high-performance liquid chromatography (HPLC) at 260 nm. **(A)** HPLC fingerprint of AWE and **(B)** of three standard references including 1: Calycosin-7-O-β-D-glucoside; 2: Ononin; 3: Calycosin; **(C)** Identification of astragaloside IV in AWE using ultra-performance liquid chromatography-tandem mass spectrometry (UPLC-MS/MS). **(D)** Structures of the parent ion and product ions of astragaloside IV.

The fingerprint of the AWE using HPLC is shown in Figure 1A. Calycosin-7-*O*-beta-D-glucoside, ononin, and calycosin were identified in the AWE sample using HPLC retention times compared with standard references (Figure 1B). The multiple reaction monitoring (MRM) scan in a positive model was utilized to detect Astragaloside IV by acquiring the sodium adduct parent ion (M + Na)^+^ to product ion transitions of *m*/*z* 807.4 → 627.5 and *m*/*z* 807.4 → 203.15 with collision voltage of 53 and 59 eV, respectively (Kafle et al., 2020). Product ion of *m*/*z* 627.5 was used for quantification. A linear calibration curve (y = 31083x + 18200, R^2^ = 0.9932) was produced using 0.625, 1.25, 2.5, 5, 10 ng/ml of Astragaloside IV. Astragaloside IV in AWE was identified according to the peak retention time and two product ion fragments in MRM channels (Figure 1C). The quantification of Astragaloside IV in the AWE was 0.048%. The structure of calycosin-7-*O*-beta-D-glucoside, ononin, calycosin, and astragaloside IV is shown in Figures 1A,D.

### 3.2 AWE Inhibits Superoxide Anion Generation and Elastase Release in Activated Human Neutrophils

**FIGURE 2:**
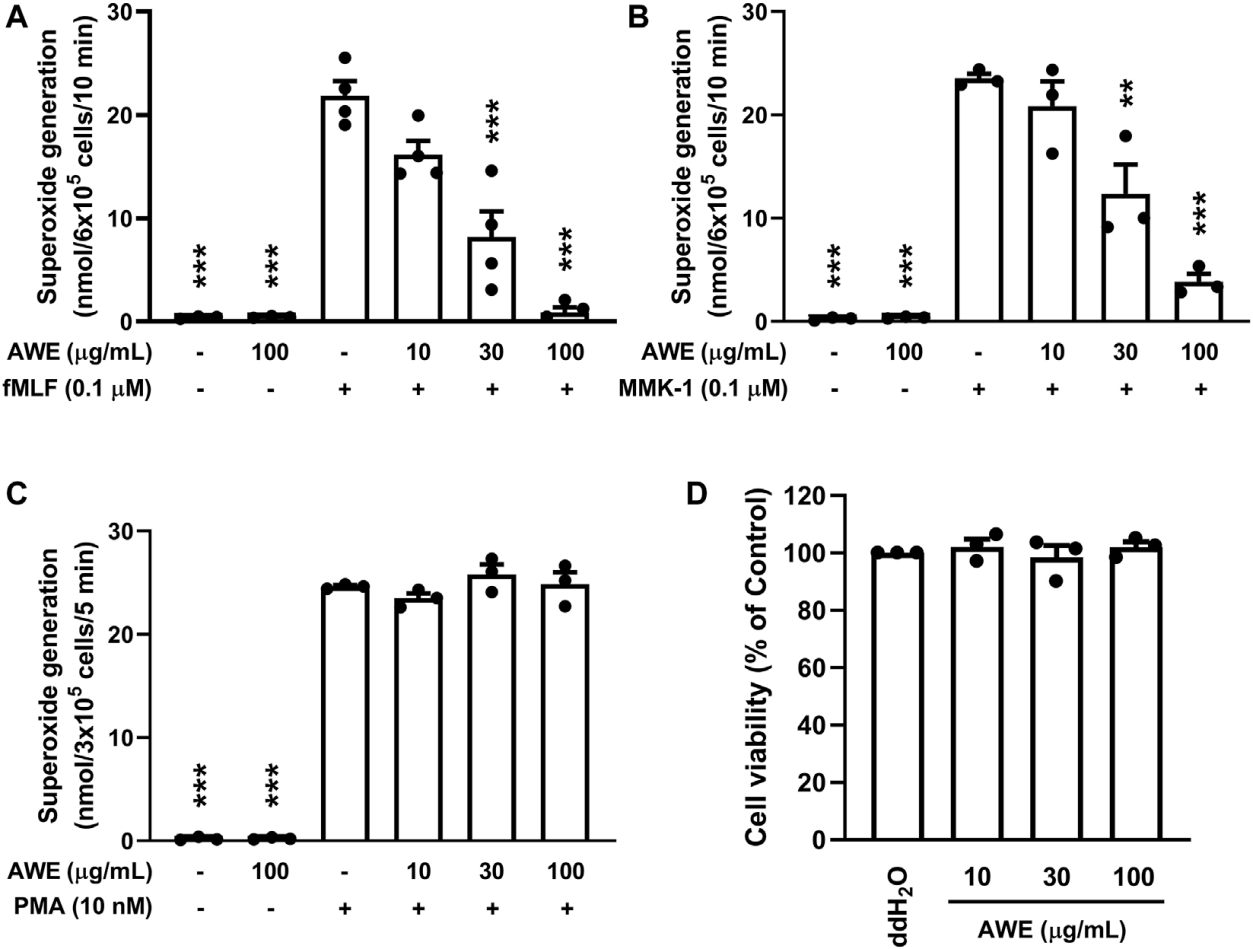
AWE inhibited superoxide anion generation in activated human neutrophils. Human neutrophils (3 × 10^5^ or 6 × 10^5^ cells/mL) were incubated with ddH_2_O (as the control) or AWE (10, 30, or 100 μg/ml) for 5 min, and then were activated by **(A)** fMLF, **(B)** MMK-1 for 10 min, or **(C)** PMA for 5 min. Superoxide anion production was monitored using ferricytochrome c reduction at 550 nm. **(D)** Human neutrophils were incubated with ddH_2_O (as the control) or different concentrations of AWE for 15 min. Cytotoxicity was represented by LDH release as a percentage of the total. The Total LDH release was determined by lysing cells with 0.1% of Triton X-100 at 37°C for 30 min. The cell viability was calculated as one minus percent of LDH release. Data are expressed as mean ± S.E.M. (*n* = 3 or 4). Statistical analysis was conducted using the one-way analysis of variance (ANOVA), followed by Tukey’s multiple comparison test if there were statistical differences in the results of ANOVA. Adjusted *p*-values of Tukey’s multiple comparison tests were defined as ***p < 0.001 and **p < 0.01 compared with the control.

Neutrophils generate a large amount of superoxide anion by activating NADPH oxidase, further creating abundant ROS by various enzymes. AWE (100 μg/ml) significantly reduced superoxide anion generation in fMLF- and MMK-1-activated human neutrophils in a dose-dependent manner (Figures 2A,B), both *p* < 0.001. Nevertheless, AWE was unable to attenuate PMA, a PKC activator, stimulated human neutrophils (Figure 2C). Moreover, we found that at 100 μg/ml concentration, AWE did not affect cell viability in the LDH releasing assay (Figure 2D).

**FIGURE 3:**
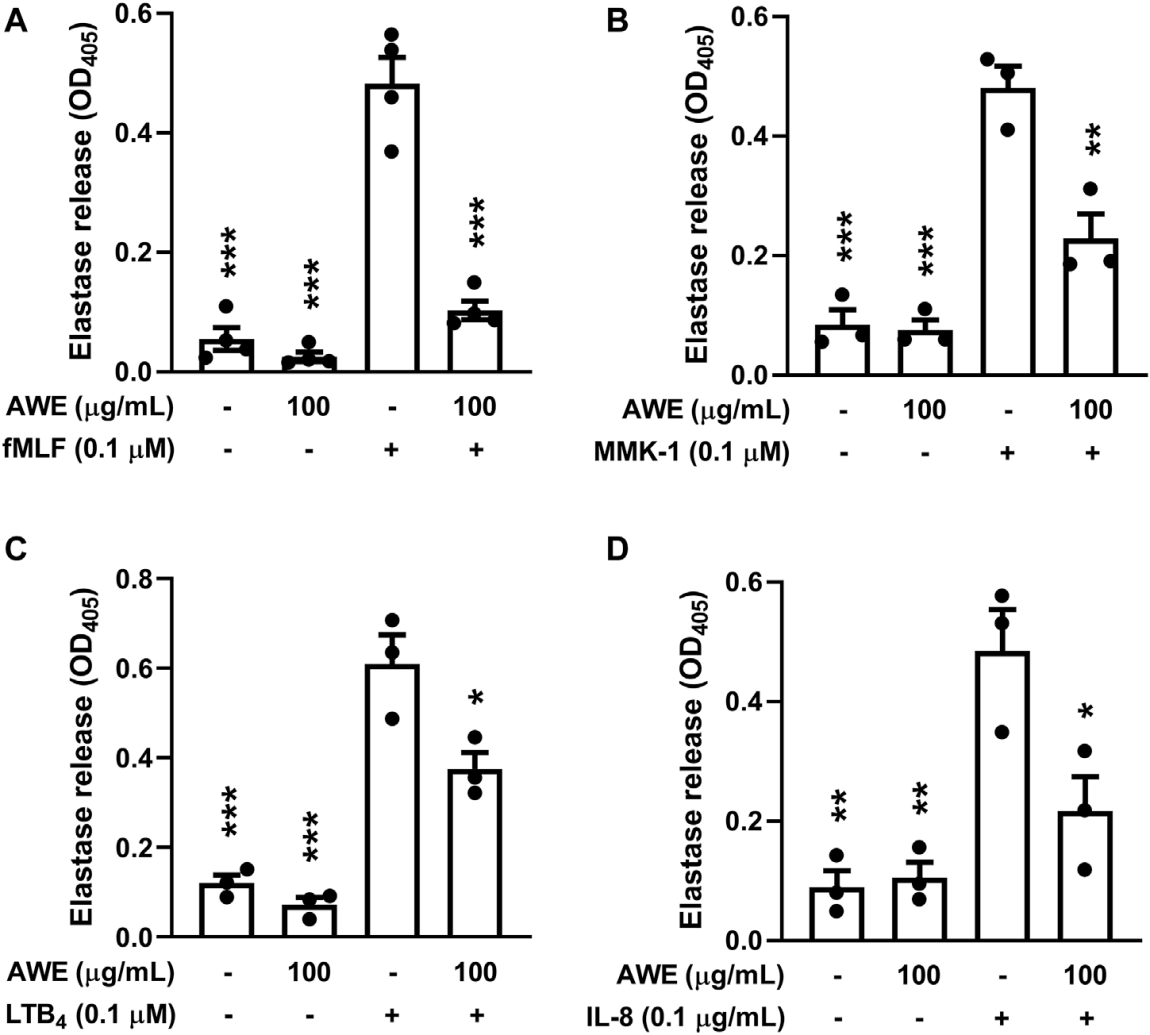
AWE inhibited elastase release in activated human neutrophils by different stimulants. Human neutrophils (6 × 10^5^ cells/mL) were incubated with ddH_2_O (as the control) or AWE (100 μg/ml) for 5 min, and then activated by **(A)** fMLF, **(B)** MMK-1, **(C)** LTB_4_, or **(D)** IL-8 and CB (0.5 μg/ml for fMLF and MMK-1 and 2 μg/ml for IL-8 and LTB_4_) for another 10 min. Elastase release was measured by spectrophotometrically at 405 nm. Data are expressed as mean ± S.E.M. (*n* = 3 or 4). Statistical analysis was conducted using the one-way analysis of variance (ANOVA), followed by Tukey’s multiple comparison test if there were statistical differences in the results of ANOVA. Adjusted *p*-values of Tukey’s multiple comparison tests were defined as ***p < 0.001, **p < 0.01, and *p < 0.05 compared with the control.

Human neutrophils create abundant granules into the cytosome, which contain numerous proteolytic and ROS-producing enzymes, including neutrophil elastase, MPO, and cathelicidin (Faurschou and Borregaard, 2003). We observed that AWE (100 μg/ml) significantly inhibited the release of neutrophil elastase in activated human neutrophils with various stimulants such as fMLF (*p* < 0.001), MMK-1 (*p* = 0.002), IL-8 (*p* = 0.0197), and LTB_4_ (*p* = 0.0121) (Figure 3). Taken together, these results indicate that AWE inhibits granule releasing and superoxide anion generation, which contributes to oxidative stress production; thus, these data supported the earlier finding that AWE reduced oxidative stress production.

### 3.3 AWE has a Substantially ROS Scavenging Effect but Neither Superoxide Anion nor Nitrogen-Centered Radicals

**FIGURE 4:**
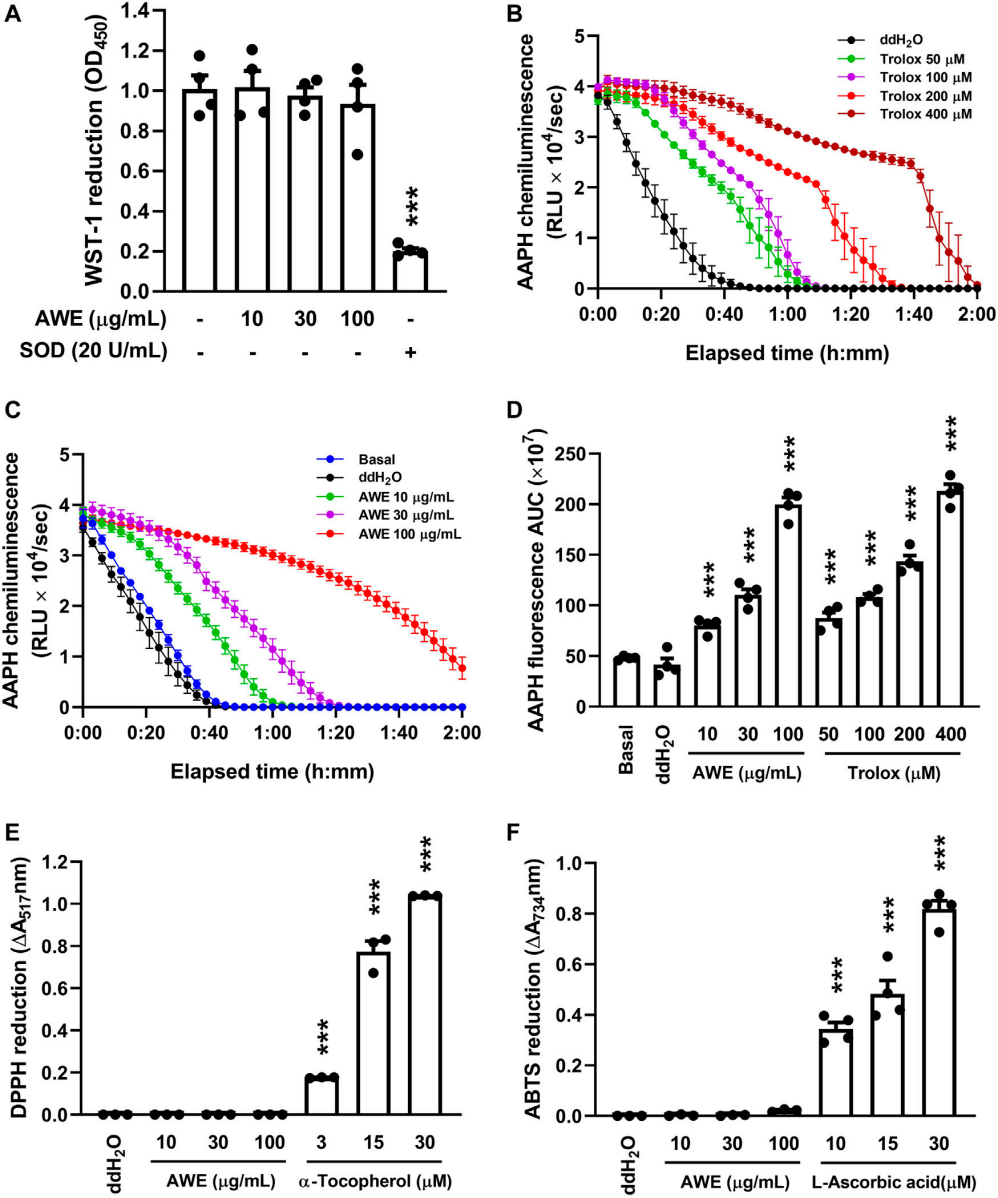
AWE has a significant ROS scavenging effect but neither superoxide anion nor nitrogen-centered radicals. **(A)** 1 mM CaCl_2_ in HBSS and xanthine oxidase were incubated with ddH_2_O (as the control). AWE (10, 30, and 100 μg/ml) was added for 3 min and then xanthine (0.1 mM) for another 10 min. Reduction of WST-1 by superoxide anion was measured spectrophotometrically at 450 nm. Superoxide dismutase (SOD) 20 U/ml was used as the positive control. Fluorescein decay curve induced by AAPH and cleared by **(B)** Trolox and **(C)** AWE in different concentrations. **(D)** The area under the curve of AWE and Trolox. **(E)** DPPH (100 μM in 99% EtOH) was incubated with ddH_2_O (as the control), AWE (10, 30, and 100 μg/ml) or α-tocopherol (3, 15, and 30 μM). The reduction of DPPH was measured spectrophotometrically at 517 nm. **(F)** ABTS (7 mM in ddH_2_O) was incubated with ddH_2_O (as the control), AWE (10, 30, and 100 μg/ml) or L-ascorbic acid (10, 15, and 30 μM). The reduction of ABTS was measured spectrophotometrically at 734 nm. Data are expressed as the mean ± S.E.M. (*n* = 3 or 4). Statistical analysis was conducted using the one-way analysis of variance (ANOVA), followed by Tukey’s multiple comparison test if there were statistical differences in the results of ANOVA. Adjusted *p*-values of Tukey’s multiple comparison tests were defined as ***p < 0.001 compared with ddH_2_O alone.

Previous studies demonstrated that AM has abundant anti-oxidative stress activity (Shahzad et al., 2016). Therefore, we used various cell-free oxidative stress generation assays to clarify the ROS scavenging ability of AWE. Figure 4A shows that AWE failed to inhibit the superoxide anion produced by the xanthine/xanthine oxidase system, with superoxide dismutase as the positive control (*p* < 0.001). This result implied that while AWE was not able to remove the superoxide anion directly, it may regulate the production of the superoxide anion in activated human neutrophils.

The clearance ability of AWE against ROS and nitrogen-centered radicals was further examined with oxygen-radical absorbance capacity assays, ABTS, and DPPH radical assays, and we observed that AWE and Trolox both had significant antioxidant effects (Figures 4B–D), as the APPH-produced ROS was cleared by AWE and Trolox to avoid the degeneration of fluorescein. However, there was no scavenging ability of nitrogen-centered radicals observed in AWE in the ABTS and DPPH assays (Figures 4E,F), in which vitamins C and E were the respective positive controls. These results suggested that AWE had a substantially better ROS scavenging ability than superoxide anion and nitrogen-centered radicals.

Taken together, our results revealed that AWE significantly reduced the oxidative stress generated by fMLF-activated human neutrophils, and this effect might have resulted from regulating the activation of human neutrophils and directly scavenging ROS by AWE.

### 3.4 AWE Reduced Neutrophil-Generated Oxidative Stress by Inhibiting Neutrophil Activation and by Scavenging ROS

**FIGURE 5:**
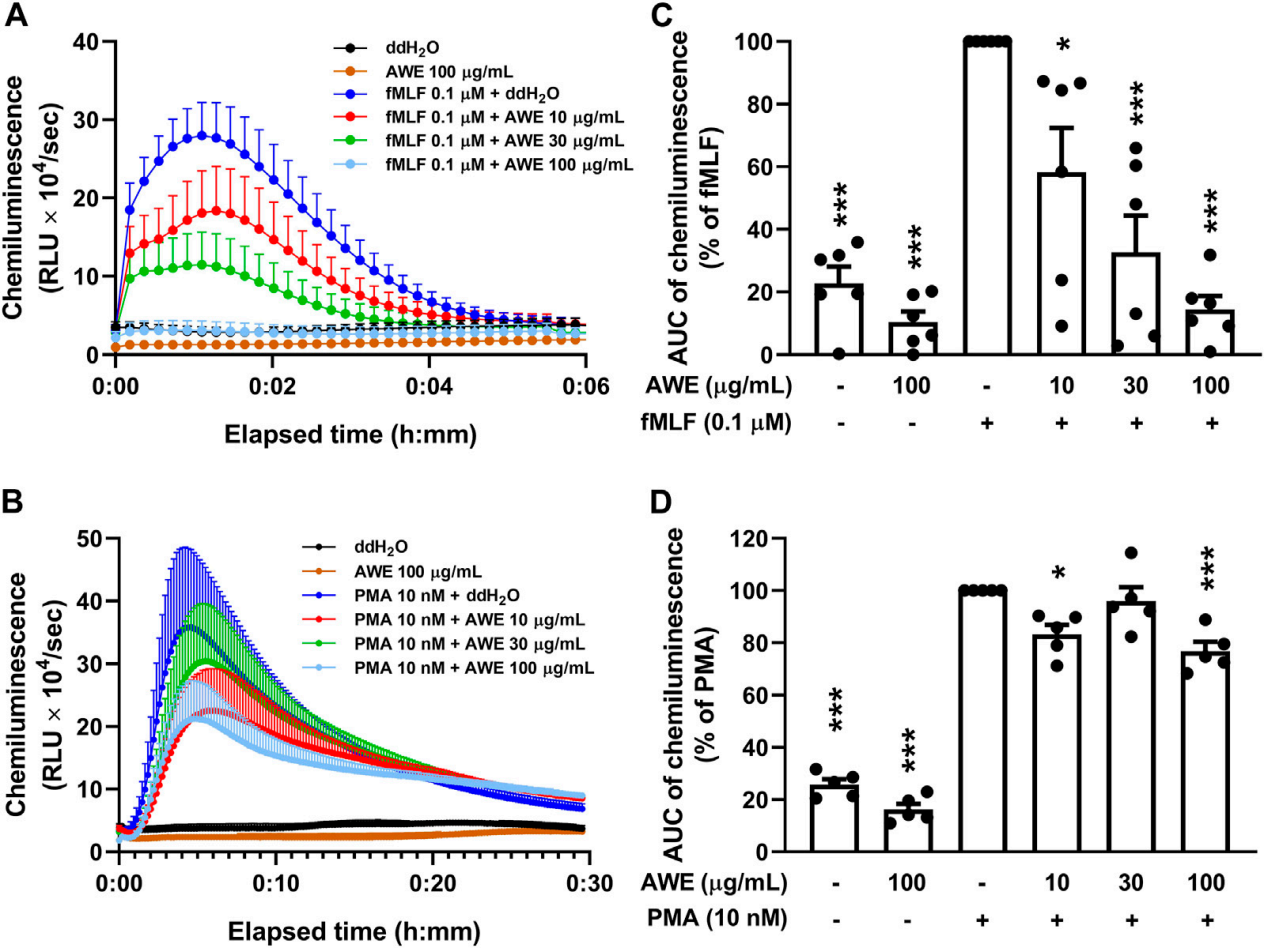
AWE both inhibited neutrophil activation and scavenged ROS generation in fMLF-activated human neutrophils in luminol-enhanced chemiluminescence assay. Human neutrophil (7 × 10^5^ cells/mL) were incubated with ddH_2_O (as the control) or AWE (10, 30, and 100 μg/ml) for 5 min and stimulated with **(A)** fMLF (0.1 μM) for another 6 min or **(B)** PMA (10 nM) for 30 min. The area under curve (AUC) of chemiluminescence in **(C)** fMLF or **(D)** PMA are shown as the mean ± S.E.M. (*n* = 5 or 6). Statistical analysis was conducted using the one-way analysis of variance (ANOVA), followed by Tukey’s multiple comparison test if there were statistical differences in the results of ANOVA. Adjusted *p*-values of Tukey’s multiple comparison tests were defined as ***p < 0.001 and *p < 0.05 compared with the control.

Intracellular and extracellular ROS were detected using luminol-enhanced chemiluminescence. AWE significantly inhibited ROS production in fMLF-activated human neutrophils at different concentrations (Figures 5A,C). The *p*-values were 0.0115, <0.001, and <0.001 for 10, 30, and 100 µg/ml. Moreover, our work revealed that AWE had a superior inhibitory effect on ROS production than on superoxide anion generation; the IC_50_ were 17.894 μg/ml and 29.539 μg/ml, respectively. Interestingly, AWE only had a minor inhibitory effect on ROS production in PMA-stimulated human neutrophils (Figures 5B,D). The *p*-values were 0.0136, 0.9461, and <0.001 for 10, 30, and 100 µg/ml. We also noted that AWE was able to reduce ROS production in resting human neutrophils without any stimulant. Taken together, these results indicate that AWE might have a direct antioxidant effect on ROS generation in activated human neutrophils.

**FIGURE 6:**
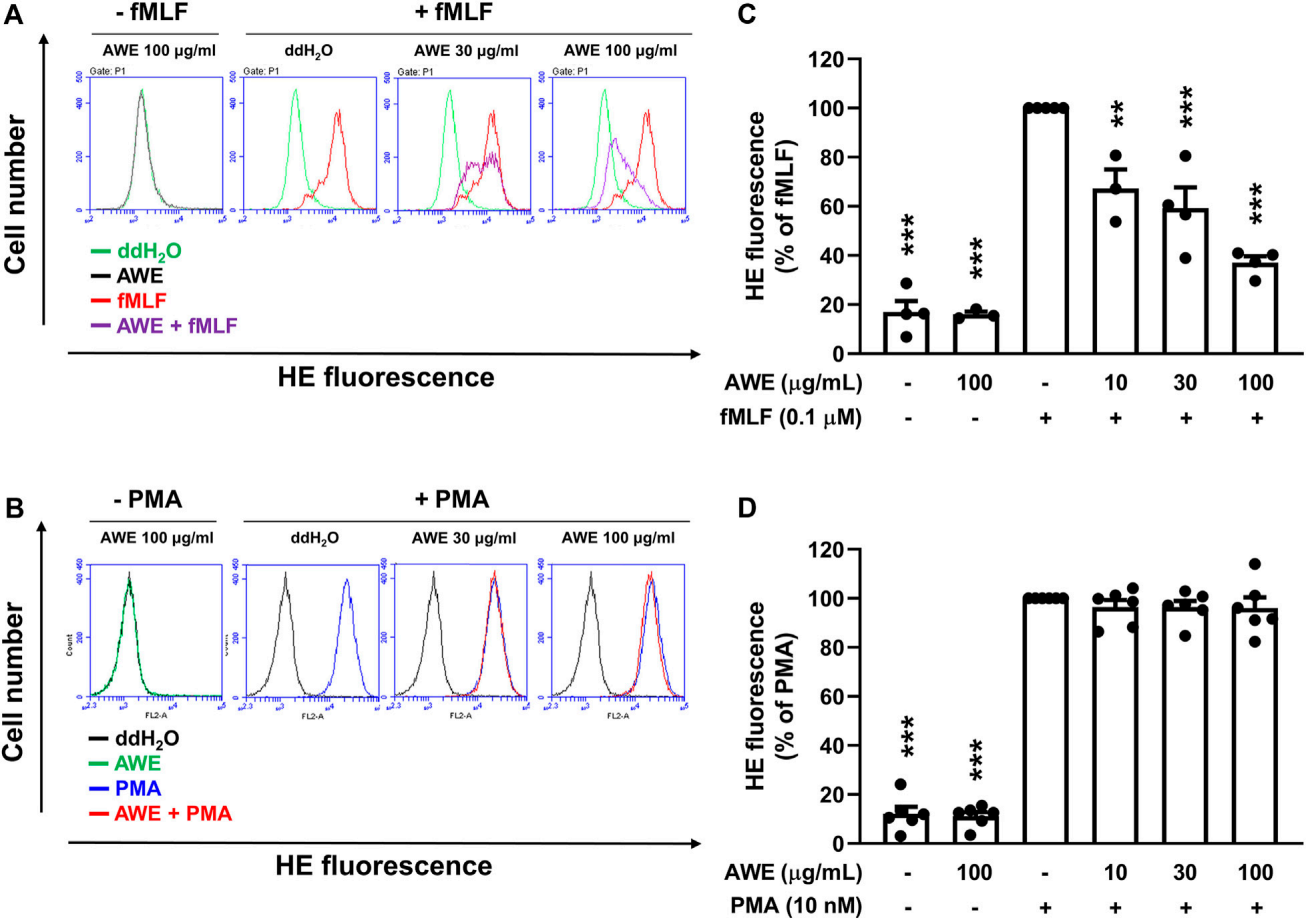
AWE inhibits intracellular superoxide anion generation in fMLF-activated human neutrophils. Human neutrophils labeled with HE were incubated with ddH_2_O (as the control) or AWE (10, 30, and 100 μg/ml) for 5 min and then stimulated with **(A)** fMLF (0.1 μM) or **(B)** PMA (10 nM) for an additional 5 min. The representative histograms demonstrating typical fluorescence-activated cell sorting profiles are shown. Mean fluorescence intensities are shown as **(C)** and **(D)**. Data are expressed as the mean ±S.E.M. (*n* = 3–6). Statistical analysis was conducted using the one-way analysis of variance (ANOVA), followed by Tukey’s multiple comparison test if there were statistical differences in the results of ANOVA. Adjusted *p*-values of Tukey’s multiple comparison tests were defined as ***p < 0.001 and **p < 0.01 compared with the control.

Since AWE showed a significantly stronger inhibitory effect on superoxide anion generation in fMLF-activated human neutrophils rather than in PMA-stimulated neutrophils, we used HE as an intracellular superoxide anion probe to clarify AWE’s inhibitory effects on human neutrophils (Laurindo et al., 2008). The result suggests that AWE significantly reduced the fluorescein in HE-labelled and fMLF-activated human neutrophils (Figures 6A,C). Consistent with our previous findings, AWE failed to inhibit the intracellular superoxide anion generation in PMA-activated human neutrophils (Figures 6B,D). Together, these findings suggest that AWE had a substantial inhibitory effect in fMLF-activated human neutrophils.

### 3.5 AWE Inhibited Activated Neutrophils From Expressing CD11b Integrin and Adhering to Endothelial Cells

**FIGURE 7:**
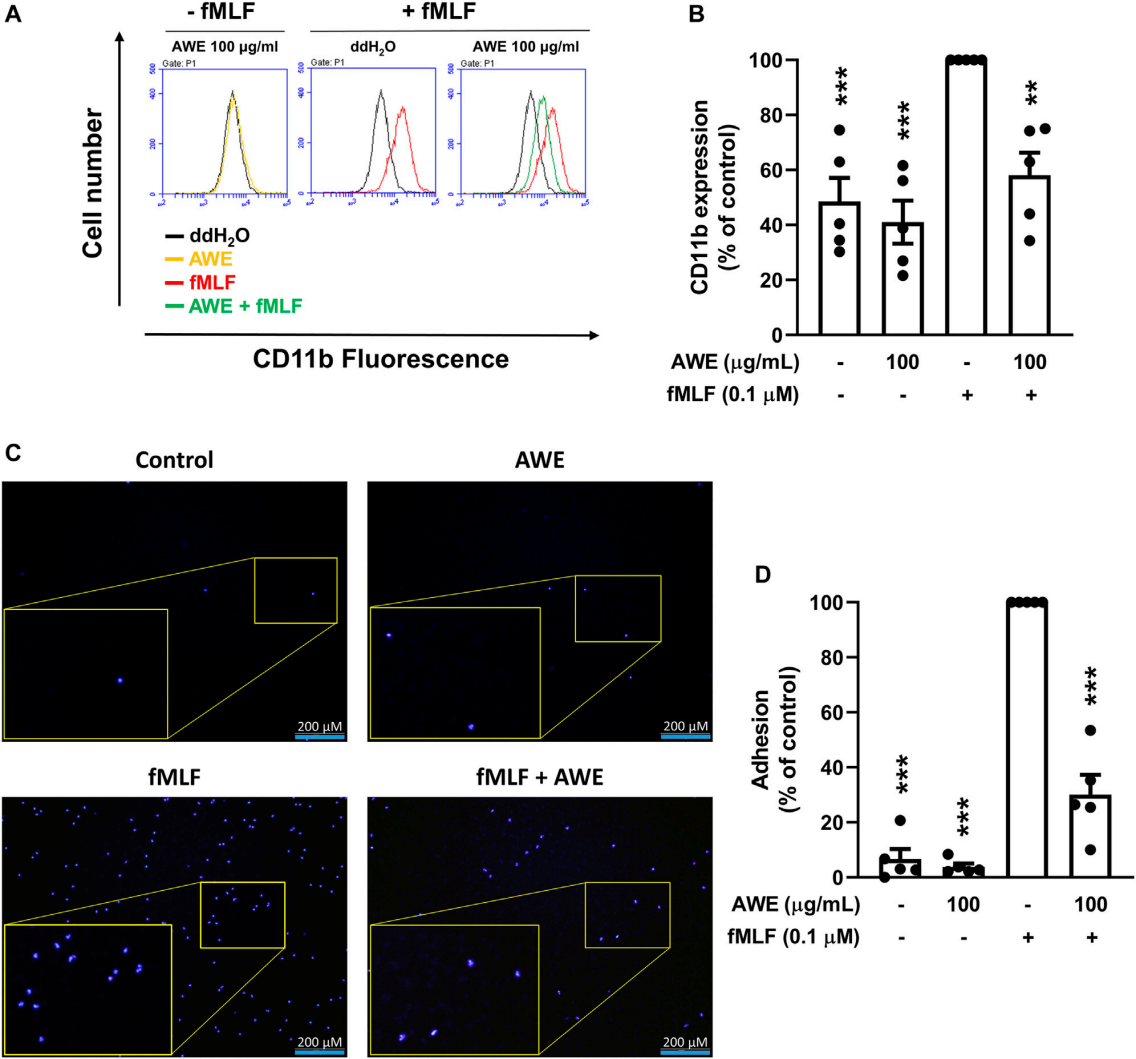
AWE suppressed the adhesion of fMLF-activated human neutrophils to bEnd.3 cells by inhibiting CD11b expression. **(A)** Human neutrophils were incubated with ddH_2_O (as the control) or AWE (100 μg/ml) for 5 min and then activated by fMLF (0.1 μM)/CB (1 μg/ml) for an additional 5 min. Representative histograms demonstrating typical fluorescence in the absence or presence of FITC-labeled anti-CD11b. **(B)** Mean fluorescence intensities are shown. **(C)** Hoechst 33342-labeled human neutrophils (4 × 10^6^ cells/mL) were incubated with ddH_2_O or AWE (100 μg/ml) for 5 min, then fMLF (0.1 μM) was used as the stimulant. The activated human neutrophils were added into bEnd.3 cells for 15 min. Adherent neutrophils on bEnd.3 cells were detected using a fluorescence microscope. Scale bar represented 200 μm. Representative histograms for fluorescent microscopy are shown. **(D)** Adherent human neutrophils were counted and quantified. Data are expressed as the mean ± S.E.M. (*n* = 5). Statistical analysis was conducted using the one-way analysis of variance (ANOVA), followed by Tukey’s multiple comparison test if there were statistical differences in the results of ANOVA. Adjusted *p*-values of Tukey’s multiple comparison tests were defined as ***p < 0.001 and **p < 0.01 compared with the control.

The neutrophil surface adhesion molecules, CD11b, were labeled with FITC and detected using flow cytometry. Human neutrophils increased the expression of CD11b following stimulation by fMLP. In contrast, AWE significantly inhibited CD11b expression in the cell membrane of activated human neutrophils (Figures 7A,B), *p* < 0.001, suggesting that AWE could decrease cell adhesion and migration in activated human neutrophils.

To ascertain whether the effects of AWE on reduction of adhesion protein can further decrease cell adhesion in activated human neutrophils, we used bEnd.3 cells to mimic the endothelial environment with fMLF-activated neutrophils. We found that AWE significantly reduced the adhesion of activated human neutrophils to bEnd.3 endothelial cells (Figures 7C,D), *p* = 0.0037. Taken together, AWE in 100 μg/ml had a significant inhibitory effect on CD11b expression and on cell adhesion in fMLF-activated human neutrophils.

### 3.6 AWE Reduces Neutrophil Infiltration and Ameliorates IMQ-Induced Psoriasis-like Skin Inflammation in Mice

**FIGURE 8:**
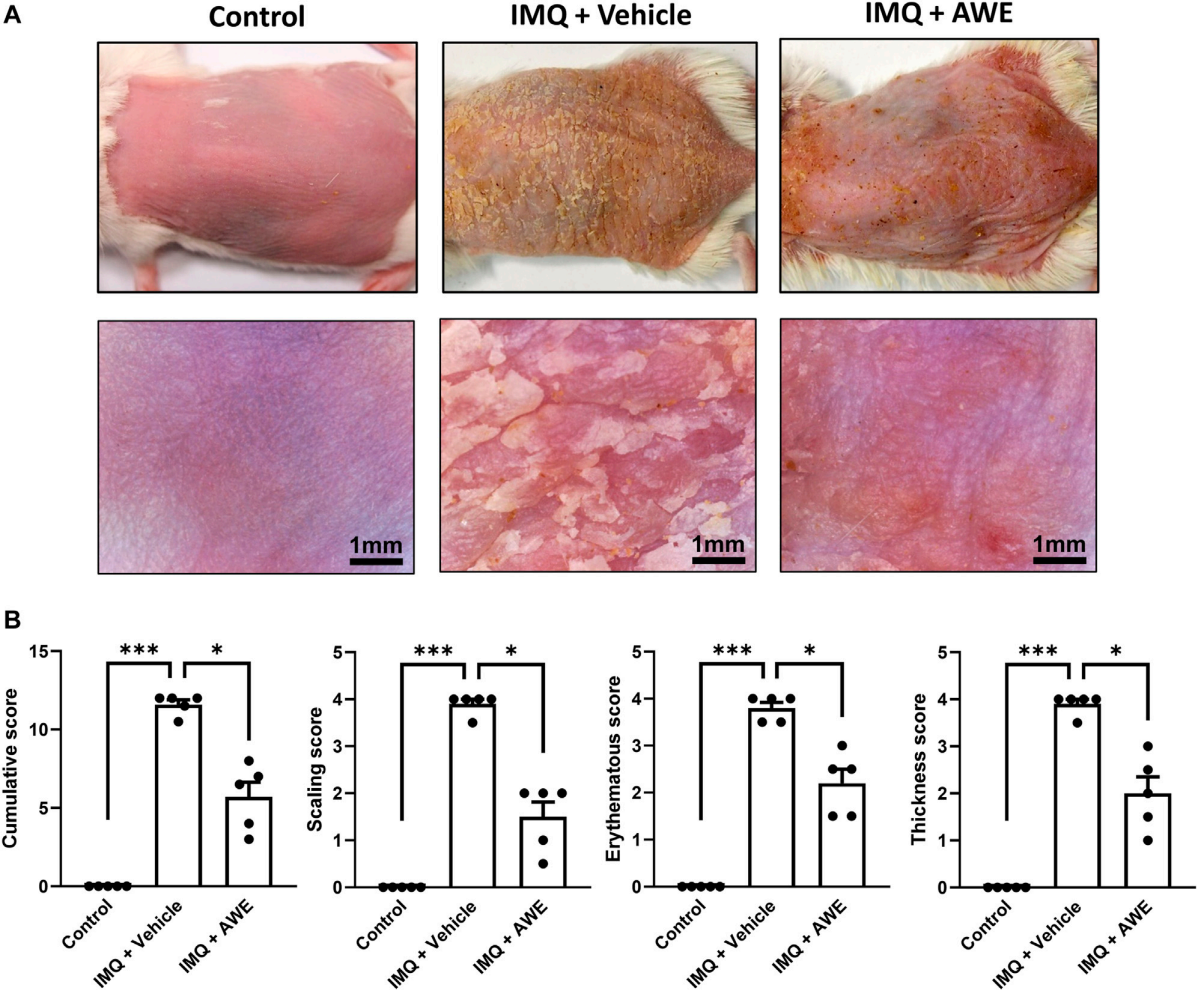
**A**WE ameliorated the severity of IMQ-induced psoriasis-like skin inflammation. **(A)** The skin change of IMQ-induced psoriasis-like skin inflammation was observed using a digital camera and a hand-held digital microscope. The scale bar of the picture in a hand-held digital microscope was 1 mm. **(B)** PASI scores, including erythema, thickness, scaling, and cumulative scores in study groups, are shown. Data are expressed as mean S.E.M. (*n* = 5). Statistical analysis was conducted using the Kruskal–Wallis test, followed by multiple comparison test with the two-stage linear step-up procedure of Benjamini, Krieger, and Yekutieli if there were statistical differences in the results of the Kruskal–Wallis test. Adjusted *q*-values of multiple comparison tests using the two-stage linear step-up procedure of Benjamini, Krieger, and Yekutieli were defined as ***q < 0.001 and *q < 0.05 compared with the IMQ plus vehicle group.

IMQ is a toll-like (TLR-7/8) receptor agonist. Application of IMQ on mice skin causes immune cell infiltration and skin inflammatory changes, such as erythema, scaling, hyperkeratosis formation, similar to those of human psoriasis (Baek et al., 2012; El Malki et al., 2013). Compared with that of the control group (without IMQ), mice treated with IMQ cream for 5 days exhibited gross skin appearance that showed psoriasis-like skin inflammation (Figure 8A). However, the topical administration of AWE alleviated psoriasis-like skin inflammation symptoms in the IMQ plus AWE group compared to that in the IMQ plus vehicle group, in which severe scaling and skin thickening were found. In addition, AWE significantly reduced the PASI score based on skin thickness (*p* = 0.0484), erythema (*p* = 0.0490), and scaling (*p* = 0.0477) in this mouse model (Figure 8B).

**FIGURE 9:**
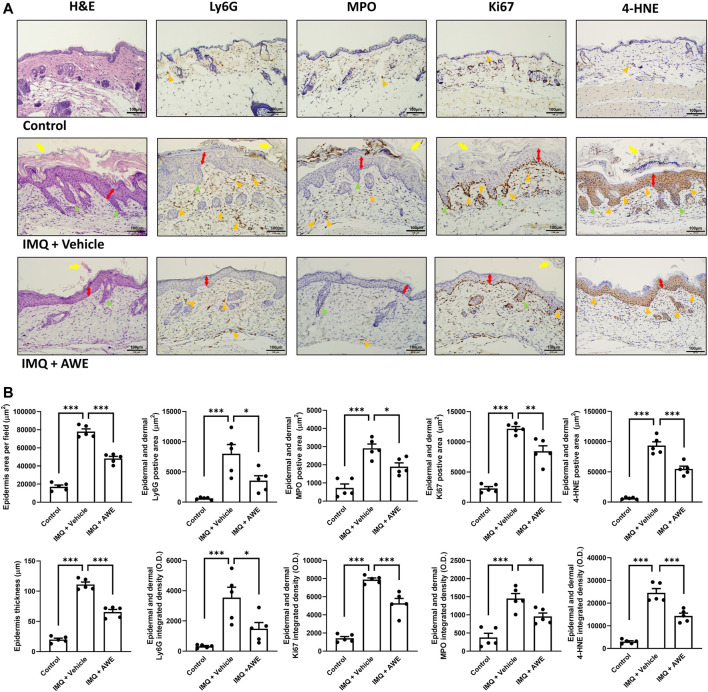
AWE reduced the epidermal thickness and neutrophil infiltration in IMQ-induced psoriasis-like skin inflammation in mice. The mice were sacrificed by deep anesthesia on day 6 after topical IMQ and AWE or vehicle treatment for 5 days. Their back skins were collected and stored in 4% formaldehyde. Hematoxylin-eosin (H&E) staining and immunohistochemical (IHC) staining for Ly6G, MPO (the neutrophil infiltration and activation marker), Ki67 (the epidermal proliferation marker), and 4-HNE (the ROS marker) were performed for all samples (marked as orange triangles). The representative images **(A)** were observed under a microscope. The scale bar was 100 μm. The IMQ plus vehicle group exhibited psoriasis-like skin inflammation. The typical histological manifestations are marked as yellow arrows (hyperkeratosis), double-sided red arrows (acanthosis), and green triangles (elongation of rete-like ridges). AWE significantly decreased neutrophil infiltration and epidermal thickness in IMQ-induced psoriasis-like skin inflammation in mice. **(B)** The quantified image data using ImageJ. Epidermal area and average epidermal thickness were calculated in H&E staining. Ly6G-, MPO-, Ki67-, and 4-HNE-positive area and integrated density were calculated in IHC staining. Data are expressed as mean S.E.M. (*n* = 5). Statistical analysis was conducted using the one-way analysis of variance (ANOVA), followed by Tukey’s multiple comparison test if there were statistical differences in the results of ANOVA. Adjusted *p*-values of Tukey’s multiple comparison tests were defined as ***p < 0.001, **p < 0.01, and *p < 0.05 compared with the IMQ plus vehicle group.

In the IMQ plus vehicle group, the IMQ-induced skin inflammation resulted in the thickened epidermis (also called acanthosis) in the H&E staining, while IHC staining revealed increased levels of Ki67, the epidermal proliferation marker. Moreover, increased Ly6G and MPO expression were observed in the IHC, indicating that neutrophils were activated and recruited from peripheral tissues. Increased 4-HNE level from lipid peroxidation implied cell damage by ROS generated by infiltrated immune cells, leading to the pathogenesis of psoriasis (Figure 9A). Aside from that, severe neutrophil infiltration was also observed in the epidermis and dermis area, which is a typical psoriasis manifestation (Munro’s microabscess). Increased thickness of the epidermis represented skin edema of psoriatic skin lesions. Hyperkeratosis and parakeratosis were also found in the epidermis area. In the IMQ plus AWE group, topical AWE (10 mg/day) significantly reduced epidermal thickness (skin edema) and hyperkeratosis compared to IMQ plus vehicle group. The IHC staining revealed a lower Ki67 positive area and integrated density (Figure 9B). In addition, lower Ly6G and MPO demonstrated that AWE inhibited neutrophil recruitment and infiltration. Reduced 4-HNE level represented decreased ROS-induced damage in the epidermis. Meanwhile, the control group had a normal epidermis and did not display neutrophil infiltration.

In summary, these results show that topical AWE administration significantly inhibited neutrophil recruitment and infiltration, thereby reducing ROS-induced damage in the epidermis and skin proliferation in the IMQ-induced psoriasis-like skin inflammation mouse model.

## 4 Discussion

Oxidative stress is caused by free radicals, such as ROS or reactive nitrogen species. Excessive ROS production, which leads to an imbalance between oxidative stress and antioxidants, causes cell and tissue damage (Xie et al., 2019). Plant polysaccharides are antioxidant constituents and abundant in natural herbal products (Jiao et al., 2016). Astragalus polysaccharides (APS), the primary components of AM, can inhibit neutrophils infiltration in the lipopolysaccharide (LPS)-induced acute lung injury rat model and reduce MPO activity and ROS level in the bronchoalveolar lavage fluid (Fang et al., 2017). A murine asthma model induced by ovalbumin-LPS demonstrated that APS inhibits neutrophil-dominant airway inflammation and how this could relate to the modulation of endoplasmic reticulum stress (Lu et al., 2016). The mechanisms underlying APS improvement of cell and tissue damage are also associated with NF-κB and p38-MAPK signaling pathways (Lu et al., 2016; Fan et al., 2019).

AM contains several bioactive flavonoids, including calycosin, calycosin-7-*O*-β-D-glucoside, ononin, and formononetin. Formononetin and calycosin, which are the non-glycoside forms of ononin and calycosin-7-*O*-β-D-glucoside, respectively (Yang et al., 2013), have a protective effect in LPS-induced cell injury and in H_2_O_2_-induced liver injury (Jiang et al., 2015; Li et al., 2015). Calycosin-7-*O*-β-D-glucoside and calycosin can scavenge nitro-oxide, resulting in tissue damage in the cerebral I/R injury rat model (Fu S. et al., 2014). Moreover, studies have shown that these isoflavones have neuroprotective effects that may involve the phosphatidylinositol 3-kinase-Akt signaling pathway (Jiang et al., 2015; Gu et al., 2021).

Astragaloside IV, an essential and influential saponin in AM, is known to have protective effects against organ and tissue injury (Li L. et al., 2017; Zhang et al., 2020). It has also been reported to exhibit ROS scavenging activity against oxidative stress, protecting ischemia/reperfusion (I/R) injury in the hearts and brain (Li H. et al., 2018; Jiang et al., 2019). Cycloastragenol, another triterpenoid saponin, is a hydrolysis product of Astragaloside IV that also has significant anti-inflammatory and antioxidant capabilities (Yu Y. et al., 2018), and is reported to play an inhibitory role in ROS-associated endoplasmic reticulum stress via NLRP3 inflammasome (Zhao et al., 2015).

These anti-oxidation components make AM an effective antioxidant that protects cells and tissues against ROS damage. Herein, AWE exhibited significant ROS scavenging capability in the AAPH cell-free system, which was compatible with other studies that showed that AM could clear ROS in H_2_O_2_ assay (Han et al., 2017; Adesso et al., 2018). Interestingly, we also found that AWE cleared neither the superoxide anion generated by xanthine oxidase nor the nitrogen-centered radicals, including DPPH and ABTS.

Neutrophils, the most plentiful leukocyte in blood, contained NADPH oxidase, which is the primary source of ROS production in the human body (Dahlgren and Karlsson, 1999; Jaquet et al., 2011; Wang et al., 2012; Ruhnau et al., 2014). Activated human neutrophils generate abundant ROS by respiratory burst, degranulation, and release neutrophil extracellular traps, causing tissue damage and aggravating many clinical diseases (Papayannopoulos, 2018).

Our study showed that AWE significantly reduced superoxide generation in fMLF-activated human neutrophils. However, even if AWE revealed a substantial ROS scavenging capability in the AAPH assay, it only showed a minor effect on clearing ROS while using luminol-enhanced chemiluminescence with or without stimulating neutrophils. Moreover, AWE did not directly scavenge superoxide anion in the cell-free xanthine/xanthine oxidase system. These results demonstrate that AWE had a significant inhibitory effect on superoxide anion generation in fMLF-activated human neutrophils.

When neutrophils are activated, granules inside the cytoplasm containing abundant proteolytic enzymes such as elastase and MPO, which contribute to a large ROS production, are released against microorganisms (Faurschou and Borregaard, 2003). Our study showed that AWE’s inhibitory effect on neutrophil activation also inhibited elastase release when neutrophils were activated by various stimulants.

Neutrophils in the peripheral blood respond to chemoattractants to infiltrate infectious or inflammatory tissues. The expression of surface adhesive molecular CD11b/CD18 and β2 integrins are well known to regulate neutrophil adhesion and transmigration (Sekheri et al., 2021). Our results also showed that AWE inhibited fMLF-activated human neutrophils by inhibiting the expression of CD11b on the cell membrane. Further, there was decreased neutrophil adherence to the vascular endothelial cell. Consistent with previous Astragaloside IV studies, we also found the same inhibitory effects on neutrophil infiltration to the inflammatory site by LPS-induced inflammatory or infectious murine model (Zhang and Frei, 2015; Huang et al., 2016), which may have resulted from the inhibition of neutrophil that express surface adherence proteins CD11b/CD18 and the downregulation of intercellular adhesion molecule-1 (ICAM-1) via NF-κB pathway (Li M. et al., 2012). In contrast, another study analyzing APS using human neutrophils and human umbilical vein endothelial cells (HUVECs) demonstrated that APS promotes the adhesion of human neutrophils to HUVECs and the upregulation of ICAM-1 on the surface of IL-1 treated endothelial cells; no effect on CD18 expression in human neutrophils treated with APS was observed (Hao et al., 2004).

Psoriasis is a chronic immune-mediated disease with erythematous, scaling, and thickening in the skin. Neutrophil infiltration causes neutrophilic inflammation, which can worsen the symptoms (Lowes et al., 2014; Chiang et al., 2019). Topical application of IMQ, a toll-like receptor 7/8 agonist, on mouse skin causes immune cell accumulation, releasing cytokine and ROS, which damages dermal and epidermal tissues, leading to psoriasis-like skin changes (Baek et al., 2012; El Malki et al., 2013). This change is due to cell proliferation in the epidermis. Ki67, a marker for cell proliferation, is overexpressed in proliferating keratinocytes in psoriatic skin, which can be regulated by T cells (Alhosaini et al., 2021). Increased IL-17-related immune responses contribute to skin cell proliferation observed in a psoriasis animal model (Nadeem et al., 2018). Although T cell and antigen-presenting cells have been known to be involved in the pathogenesis of psoriasis, neutrophils play a critical role by infiltrating the epidermis and secreting copious amounts of IL-17A, thus affecting keratinocytes and leading to skin proliferation (Katayama, 2018). In this study, we found that in the IMQ-induced psoriasis-like skin inflammation model, the topical application of AWE significantly improved skin inflammation and both the individual and cumulative PASI scores. Moreover, AWE reduced dermal thickness, neutrophil infiltration, MPO release, ROS-induced damage, and skin proliferation, as observed in microscopic IHC-stained slides. These results are consistent with a recent study of cycloastragenol on IMQ-induced psoriasis-like skin lesions, which regulate macrophage via inhibiting NLRP3 inflammasome-mediated pyroptosis (Deng et al., 2019).

In the pathogenesis of psoriasis, multiple immune cell interactions lead to skin proliferation and hyperkeratosis. Resident macrophages also play an important role in releasing chemoattractants to recruit neutrophils from peripheral blood to the inflammatory site. It is not clear whether the inhibitory effect of AWE on neutrophil recruitment in this animal model is associated with the inhibition of macrophage activation or not. However, studies have found that APS and astragaloside IV could activate isolated mouse peritoneal macrophages and RAW 264.7 through the NF-κB pathway (Lee and Jeon, 2005; Li Y. et al., 2017; Wang et al., 2017; Li ZP. et al., 2018). In different cancer cell lines, such as breast cancer (Li et al., 2019), lung cancer (Bamodu et al., 2019), and liver cancer (Yu J. et al., 2018), APS exerts anti-tumor effects by activating macrophages (Liu et al., 2017; Li et al., 2020). Moreover, APS has been found to promote alveolar macrophage phagocytosis and decrease lung inflammation in a chronic obstructive pulmonary disease animal model (Chu et al., 2016). Interestingly, the flavonoids and saponin in *Astragalus* also exerted an inhibitory effect on LPS-stimulated RAW 264.7 macrophages and mouse peritoneal macrophages via the NF-κB pathway (Wang et al., 2002; Lee DY. et al., 2013; Li J. et al., 2018). The inhibitory effect of astragaloside IV on macrophages via the NF-κB pathway has also been reported in a mouse abdominal aortic aneurysm model (Wang et al., 2018). Another rat dialysis peritoneal fibrosis model demonstrated that *Astragalus* extract inhibited macrophage recruitment and activation (Li et al., 2014).

Some studies on astragaloside IV have reported that it promotes macrophages M2 polarization to reduce the inflammatory state and increase tissue healing in cerebral ischemia–reperfusion injury and inflammatory bowel disease animal models (Li et al., 2021; Tian et al., 2021). Nevertheless, another study on the effect of astragaloside IV in lung cancer showed a decrease in M2 macrophages to reduce tumor growth and metastasis (Xu et al., 2018).

Despite the inconsistent results of studies on the effect of *Astragalus* on the macrophages, we focused on the effect of AWE on neutrophils and found the inhibitory effect on neutrophil recruitment and activation in psoriasis animal models. However, our limited results could not rule out the possible effect of AWE on macrophages in psoriasis. Further studies are needed to determine the promoting or inhibitory effect of the different components in AWE on macrophages.

In this study, AWE showed its antioxidant capability of ROS scavenge and directly inhibited neutrophil activation, further ameliorating neutrophilic inflammation in IMQ-induced psoriasis-like skin lesions in mice. We also identified active components in AWE, including calycosin, ononin, calycosin-7-*O*-β-D-glucoside, and Astragaloside IV. Since APS, flavonoids, and saponins in AM have anti-inflammatory bioactivities, further research is warranted to clarify whether the anti-inflammatory effects observed were attributed to individual components or combination effects. Furthermore, research targeting the signaling pathway on inhibiting activation of neutrophils by AWE could promote developing anti-neutrophilic inflammation drugs.

## 5 Conclusion

In summary, our work demonstrated the direct inhibitory effect of AWE on fMLF-activated human neutrophils, which also inhibited superoxide generation and elastase release. Moreover, AWE effectively scavenged ROS in the AAPH assay and directly inhibited neutrophil activation, further reducing CD11b expression and adhesion to vascular endothelial cells. AWE also ameliorated skin lesions in IMQ-induced psoriasis-like skin inflammation and inhibited neutrophil infiltration, MPO release, ROS-induced damage, and skin proliferation. Our results suggest that AWE can potentially inhibit neutrophil activation and alleviate neutrophilic inflammation in psoriasis.

## Data Availability

The original contributions presented in the study are included in the article/Supplementary Material, further inquiries can be directed to the corresponding authors.
