# Supplementary figures and images for: Astragalus mongholicus Bunge Water Extract Exhibits Anti-inflammatory Effects in Human Neutrophils and Alleviates Imiquimod-Induced Psoriasis-Like Skin Inflammation in Mice
Source: Front Pharmacol. 2021 Dec 10;12:762829. doi: 10.3389/fphar.2021.762829 (PMC8707293; doi:10.3389/fphar.2021.762829)

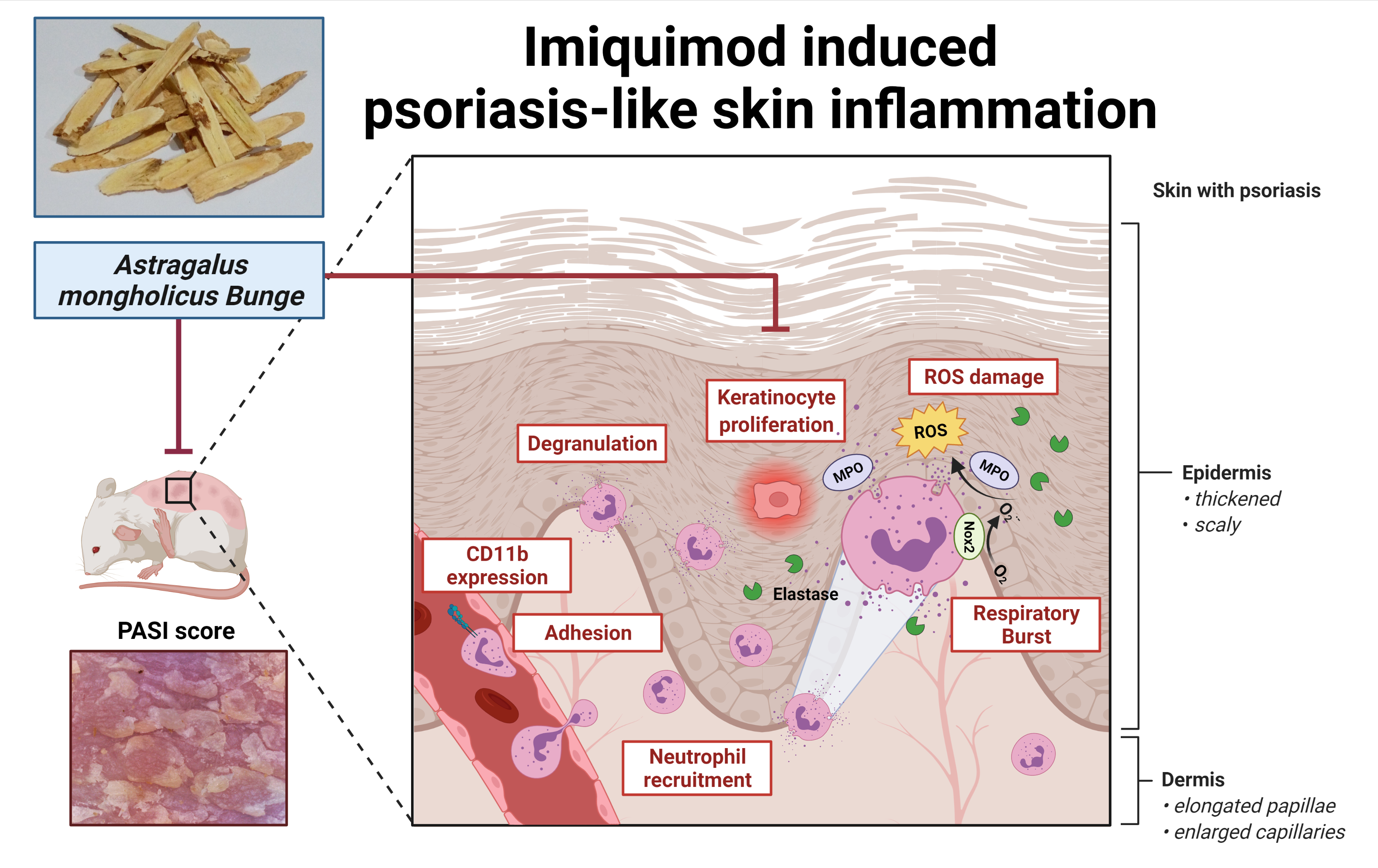

Supplement: Supplementary file 1 [file Image1.JPEG]
